# The Effect of Oblique Lumbar Interbody Fusion Compared with Transforaminal Lumbar Interbody Fusion Combined with Enhanced Recovery after Surgery Program on Patients with Lumbar Degenerative Disease at Short-Term Follow-Up

**DOI:** 10.1155/2021/5806066

**Published:** 2021-09-27

**Authors:** Wenxiang Chen, Huiying Yang, Xuesheng Jiang, Shunwu Fan

**Affiliations:** ^1^Department of Orthopaedics, Huzhou Central Hospital, Affiliated Central Hospital Huzhou University, Huzhou, Zhejiang, China; ^2^Department of Orthopedics, Sir Run Run Shaw Hospital, Zhejiang University, Hangzhou, Zhejiang, China

## Abstract

**Purpose:**

Oblique lumbar interbody fusion (OLIF) approach has been increasingly frequently performed in recent years. However, neither studies of OLIF approach nor the researches of the application of enhanced recovery after surgery (ERAS) in spinal surgery are relatively rare. Here, our study is aimed at investigating the therapeutic effects of the application of OLIF compared with transforaminal lumbar interbody fusion (TLIF) approach combined with ERAS in dealing with this disorder at short-term follow-up. *Material and Methods*. Thirty-eight patients who undergone OLIF and forty patients who undergone TLIF with pedicle screws were included in our study. The concept of ERAS was applied in the perioperative period of the patients. Preoperative and postoperative laboratory test indexes of blood were examined and evaluated in all individuals. Visual analogue scale (VAS), Oswestry disability index (ODI), and Clinical Symptom Score of the Japanese Orthopaedic Association (JOA) were used in preoperative evaluation and postoperative follow-up. Satisfaction survey was also performed after surgery.

**Result:**

The postoperative results of red blood count, C-reaction protein, D-dimer, and albumin were still within the reference ranges in most of the patients. It was shown that objective evaluations including VAS score, ODI index, and JOA score were significantly improved after OLIF and TLIF surgery. The follow-up of 6 months after surgery showed that VAS, ODI, and JOA were improved more in the OLIF group than that in the TLIF group. The overall satisfaction (satisfied and very satisfied) was 95% and 97.4% in the TLIF group and the OLIF group, respectively, and there was no difference between the two groups.

**Conclusion:**

This study indicated that OLIF and TLIF approach were both rather effective therapies for patients with lumbar degenerative diseases. The effect of OLIF procedure could be better than TLIF procedure in the early stage after surgery.

## 1. Introduction

Lumbar degenerative disease happens mainly on account of gradual degeneration of intervertebral disk with increasing age, which consists of lumbar disc herniation, lumbar spinal stenosis, lumbar degenerative slippage, lumbar spondylolysis with or without vertebral slippage, lumbar degenerative scoliosis, and discogenic lumbago [1, 2]. Patients underwent surgical treatment after ineffective conservative therapy. More and more patients need surgery, and the costs of surgery are continuously increased [3]. It leads to heavy economic and social burdens by surgical treatment. The total annual direct expense of lumbar spinal fusion has risen nearly 8-fold to $33.9 billion in the United States from 1998 to 2008 [4].

Lumbar interbody fusion (LIF) is a determined treatment for lumbar degenerative disease [5]. LIF is commonly performed by using five main approaches consisting of posterior lumbar interbody fusion (PLIF), transforaminal lumbar interbody fusion (TLIF), lateral lumbar interbody fusion (LLIF), oblique lumbar interbody fusion (OLIF), and anterior lumbar interbody fusion (ALIF) [6]. Traditional posterior approaches are limited by causing iatrogenic injury to the paraspinal musculature and disruption of the posterior tension band [7] The rate of abdominal and vascular complications is common with anterior approach [8]. In contrast, advantages of OLIF approach are apparent. As a minimally invasive surgery, OLIF approach can obtain rapid postoperative mobilization. Otherwise, the interbody fusion cage of OLIF approach is bigger than those of other approaches; thus, the fusion rate is higher [9]. Lumbar plexus and psoas injury are rare because the incision carried out anterior to the psoas [10].

Enhanced recovery after surgery (ERAS) programs have become increasingly popular in different fields of surgery over the past decade [11]. The concept of ERAS was originally put forward in the field of general surgery and colonic resection [12]. The main principle of ERAS is to decrease trauma and stress by using the least surgical invasion, thereby to reduce postoperative complications, to shorten length of stay, to increase patient satisfaction, and to obtain a faster recovery. The application of ERAS plays a crucial role in better treatment effects after surgery [13, 14].

OLIF approach has been increasingly frequently performed in recent years. However, neither studies of OLIF approach nor researches of the application of ERAS in spinal surgery are relatively rare. Therefore, our study is aimed at investigating the therapeutic effect of the application of OLIF approach compared with TLIF procedure combined with ERAS in dealing with lumbar degenerative disease.

## 2. Methods

### 2.1. Participants

We included 38 patients who undergone OLIF and 40 patients who undergone TLIF with pedicle screws in our hospital between December 2015 and December 2017. All patients selected should conform to the following criteria. Inclusion criteria were as follows: (1) patients diagnosed with lumbar disc herniation, lumbar spinal stenosis, degenerative slippage I-II degrees, and lumbar spondylolysis with/without vertebral slippage I-II degrees; (2) the lumbar vertebral degeneration mainly located in L4/L5; (3) symptoms included chronic low back pain with or without lower limb radiating pain (unilateral or bilateral), for whom conservative treatments had no effect within at least 6 months; (4) patients were performed with a single-level surgery of L4/L5. Exclusion criteria were as follows: (1) lumbar intervertebral disc herniation with fibrous ring rupture or nucleus pulposus dissociation; (2) severe lumbar spinal canal stenosis required direct decompression of the vertebral canal; (3) spontaneous fusion of lumbar joint in the lesion segment; (4) peritoneal surgery or spine surgery was performed before; (5) diagnosed with severe osteoporosis; (6) patients were undergone multilevel surgery. The follow-up time was six months and twelve months. Our study was approved by the institutional review boards of the hospital, and informed consent was obtained from all study participants according to the Helsinki Declaration.

### 2.2. ERAS Protocol

Our ERAS interventions were divided into preoperative, intraoperative, and postoperative and included administration of the following: (1) patient education and counseling, (2) preoperative fasting, (3) antibiosis before surgery, (4) standard anesthetic protocol, (5) multimodal analgesia, (6) early feeding after surgery, (7) gastrointestinal management, (8) early mobilization medical, (9) early removal of bladder catheter, and (10) antithrombotic prophylaxis.

### 2.3. Preoperative Management

Preoperative blood routine examinations, blood biochemistry, conventional coagulation examinations, immunology tests, urine routine examinations, and stool routine examinations were all performed. Most patients were examined with anteroposterior and lateral radiographs of the spine, hyperflexion, and hyperextension radiographs of the lumbar spine, lumbar CT, and lumbar MRI. If the patients had other specific diseases, we would determine what should be checked according to the diseases. The conditions of some patients with chronic diseases, such as hypertension or diabetes, were adjusted to the normal range or the tolerant ranges of the operation. We visited patients repeatedly, answered their questions carefully, and comforted them to relieve their stress reaction and anxiety. The surgical site was marked before the patient entered the operation room.

### 2.4. Procedures

The patients who underwent OLIF surgery were operated by three senior spinal surgeons. Two of them assisted the surgeon who performed all the surgeries all the time. The patient was placed on the right side position, and the surgical incision was on the left side. The incision of the single segment was made by extending 2-3 cm from the midpoint of the target intervertebral space to the ventral side. The skin and subcutaneous tissues were incised; the muscle fascia was incised along the direction of the external oblique, the internal oblique, and the transverse abdominal muscle, respectively, followed by blunt separation along the direction of the muscle fiber and entering the retroperitoneal space. The lumbar and abdominal aortic spaces were exposed under direct vision. Abdominal organs, vascular sheath, ureter, peritoneal membrane, and other tissues were pulled to the abdomen side. Lumbar maximus muscle was pulled to the dorsal side by clinging to the surface of the intervertebral disc. The guide needle was inserted after exposing the target intervertebral disc. The correct operative segment was determined by “C” arm X-ray machine, and the guide needle was adjusted to the middle of the intervertebral space. Then, the expansion sleeve and the channel with the light source were inserted. The sleeve was removed, and the channel was fixed, and a perfect surgical field of vision was exposed. The target intervertebral disc was removed, then the cartilaginous endplate was removed. The intervertebral space was opened using a test mode sequentially. We selected the appropriate height and length of the fusion device filled with the artificial bone or autologous iliac bone and knocked it into the intervertebral space. During the operation course of the model test and fusion device implantation, it was important to enter along the oblique line first and then rotate it to enter vertically into the intervertebral space. The key procedure throughout the operation relied on a fluoroscopy to determine that the test was always located in the middle of the intervertebral space. The left and right of the test could be placed on the epiphyseal rings around the periphery of the vertebral body. Finally, the retractor system was removed carefully. The adjacent soft tissue, such as blood vessels and nerves, was examined again. The surgical area was washed with saline irrigation and iodophor, then the incision was sutured layer by layer after bleeding ceased. One drainage tube was placed in the surgical area. A device of pedicle screw-rod internal fixation was performed through the interstitial approach of multiple fissures and the longest muscle.

The patients who underwent TLIF surgery were operated by three senior spinal surgeons. Two of them assisted the surgeon who performed all the surgeries all the time. A single-level instrumented TLIF was performed with polyaxial pedicle screws and crescent-shaped interbody cage. Decompression of lateral recess/foramina was performed in case of obviously stenosis. Osteotomy of the endplates was performed in anterior portions of endplates followed by insertion of morcellized bone obtained by approach/decompression. Interbody cage of maximal feasible height was placed posterior to bone grafts. The technique was performed in the standard manner.

### 2.5. Postoperative Management

Antibiotics were given to prevent infection, and analgesic drugs were given to relieve pain after surgery. Postoperative blood routine examinations, blood biochemistry, and conventional coagulation examinations were also performed. Patients were required to perform a reexamination of anteroposterior and lateral radiograph of the spine, lumbar CT, and lumbar MRI. On the basis of the examination results, we took an appropriate treatment for the patients. The drainage catheter of the incision and catheters was removed within 48 hours after surgery. Then, the patients wearing waist circumference could get out of bed and walk in the ward under the guidance and assistance of medical staffs. The frequency of dress changing for the wounded was about once/2-4 days, and the stitches would be removed about 2 weeks after surgery. We visited patients repeatedly, inquired their conditions of postoperative recovery, and encouraged them to take an active rehabilitation exercise. Generally, the patient was discharged 3-5 days after surgery and was informed that wearing waist circumference was necessary, and twisting or bending waist was forbidden in 3 months.

### 2.6. Evaluation Method and Observation Index

The operative duration, blood loss, hospital stays, and complications during and after operations were recorded. The visual analogue scale (VAS), the Oswestry disability index (ODI), and Clinical Symptom Score of the Japanese Orthopaedic Association (JOA) were used in preoperative evaluation and postoperative follow-up. Satisfaction survey was also performed twelve months after surgery.

### 2.7. Statistical Analysis

Statistical analyses were performed using Prism 7 (GraphPad Software, Inc., San Diego, CA, USA). The results are expressed as mean ± SD. Statistical differences were assessed by Student's *t* test, nonparametric tests, or one-way ANOVA followed by Tukey's post hoc analysis. Values of *p* < 0.05 were indicated statistically significant.

## 3. Results

### 3.1. General Characteristics of Patients

Age, gender, and BMI of the patients in the TLIF group and the OLIF group had no significant difference. Eleven cases and eight cases had smoking history in the TLIF group and the OLIF group, respectively. The total duration of operation had no difference between the TLIF group and the OLIF group. The intraoperative blood loss was remarkably less in the OLIF group compared with the TLIF group. In addition, the duration of hospital stay was obviously shorter in the OLIF group compared with the TLIF group (Table 1).

### 3.2. Preoperative and Postoperative Laboratory Test Index of Blood

**Figure 1:**
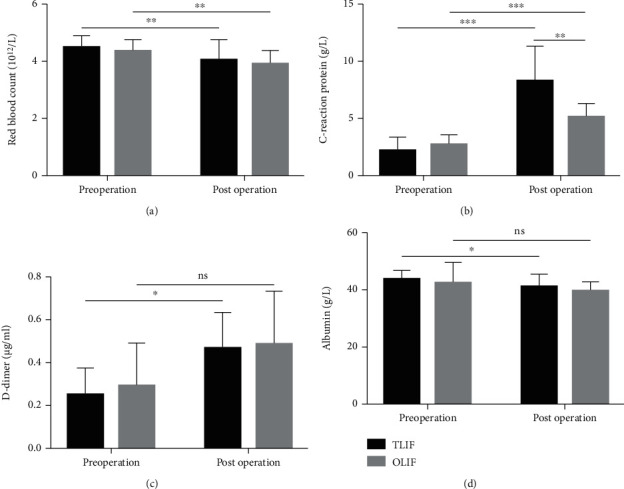
The values of red blood count (a), C-reaction protein (b), D-dimer (c), and albumin (d) in the TLIF and OLIF group preoperatively and the first day after surgery. OLIF: oblique lumbar interbody fusion; TLIF: transforaminal lumbar interbody fusion. Data is presented as mean ± SD. ns: no significance, ^∗^*p* < 0.05, ^∗∗^*p* < 0.01, ^∗∗∗^*p* < 0.001, compared with the TLIF group.

Preoperative and postoperative blood parameters could reflect blood loss, the risk of thrombus formation, inflammation, and nutritional status in perioperative patients. It was shown that the values of postoperative red blood count and albumin were reduced, and the values of C-reaction protein and D-dimer were increased compared with those of the preoperative index in patients who undergone TLIF or OLIF. The value of CRP in the OLIF group was significantly lower than that in the TLIF group on the first day postoperatively. However, the postoperative results of red blood count, C-reaction protein, D-dimer, and albumin were still within the reference ranges in most of the patients (Figure 1). Taken together, the application of ERAS in the minimal invasive surgery including OLIF and TLIF could remarkably reduce blood loss, inflammatory reaction, the risk of thrombus formation, and nutrient loss.

### 3.3. Clinical Outcome and Complications

**Figure 2:**
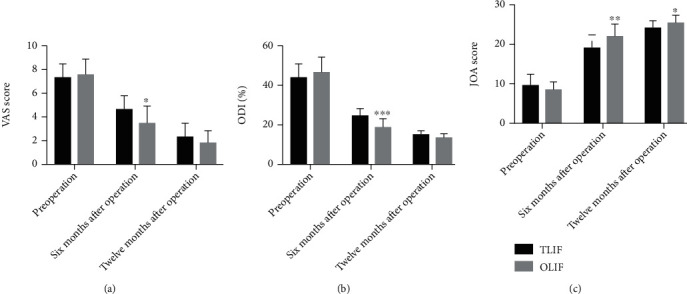
VAS score (a), ODI index (b), and JOA score (c) in the TLIF and OLIF group preoperatively and six and twelve months after surgery. Data is presented as mean ± SD. ^∗^*p* < 0.05, ^∗∗^*p* < 0.01, ^∗∗∗^*p* < 0.001, compared with the TLIF group.

The VAS score and ODI index of six and twelve months after operation were significantly lower than those of preoperation in the TLIF group and the OLIF group, while the JOA score of six and twelve months after operation was significantly higher than that of preoperation in the TLIF group and the OLIF group (Table 2). The VAS score and ODI index score of six months after operation in the OLIF group were obviously lower than those in the TLIF group, while the JOA score of six and twelve months after operation in the TLIF group was significantly higher than that in the OLIF group. However, the VAS score and ODI index of twelve months after operation had no significant difference between the OLIF group and the TLIF group (Figure 2). Furthermore, the overall satisfaction (satisfied and very satisfied) was 95% and 97.4% in the TLIF group and the OLIF group, respectively, and there was no difference between the two groups (Table 3). Taken together, it was shown that objective evaluation including VAS score, ODI index, and JOA score was significantly improved after OLIF and TLIF surgery, and the result of satisfaction survey, a representation of subjective assessment, was almost satisfied, and the results in the OLIF group were slightly better than those in the TLIF group.

## 4. Discussions

This study investigated the curative effects of patients with lumbar degenerative diseases by applying OLIF or TLIF approach combined with enhanced recovery program. Postoperative laboratory test index of blood showed patients were with less blood loss, no infection, no thrombus formation, and little change in nutritional status. Early removal of the urinary catheter and drainage tube was conducive to leave the bed to perform functional exercise in our patients. The pain was alleviated noticeably, and lower limb muscle strength and sensory function were obviously improved in our patients. The results of postoperative follow-up showed that the VAS score, ODI index, and JOA score were significantly improved compared with those of preoperation in all the participants. The overall satisfaction (satisfied and very satisfied) was relatively good in the TLIF group and the OLIF group.

The concept of ERAS has been widely used in many surgical procedures including spine surgery. Compared with the traditional treatment groups, the treatment groups with the concept of ERAS have better postoperative indexes, such as improvement of symptom and rapid recovery from surgical trauma, which undoubtedly reduces the economic and social burden on patients and their families. In this study, we introduced the concepts of ERAS, which included no pain, no infection, no thrombus, no tube, intensive nourishment, and early postoperative rehabilitation. Besides, we put more emphasis on the psychological condition of the patients before and after the operation because psychological factor played a crucial role in postoperative rehabilitation [15, 16]. We reported the advantages of OLIF surgery combined with ERAS in the treatment of spinal degenerative diseases for the first time. Some other researches about applying ERAS get similar conclusions to ours. For example, Wang et al. [17] found ERAS programs for spinal fusion surgery decreased the costs of acute care owing to less invasive interventions to minimize soft tissue damage. Their another study [18] suggested that ERAS combined lumbar fusion surgery reduced postoperative recovery time and complications.

Despite different lumbar interbody fusions have different strengths and limitations, respectively, OLIF approach has been widely accepted and performed more frequently due to its minimally invasive approach, fewer complications, and a shorter learning curve in recent several years. Zairi et al. [19] reported no vascular injuries or peripheral nerve trauma happened, which were benefited from the surgical procedure in their study. Similarly, another study showed that the OLIF technique reduced approach-related perioperative comorbidities of L4/L5 level diseases by eliminating muscle and nerve manipulations [20]. Additionally, a case report also suggested that using the minimally invasive OLIF procedure including L5-S1 fusion showed a great superiority in dealing with degenerative kyphoscoliosis in a patient with Parkinson's disease because of its less invasive approach [21]. However, some complications of OLIF approach including vascular and nerve injury could not be ignored. The position should be as close as possible to the proximal intervertebral disc of the proximal intervertebral body which can effectively reduce the risk of segmental artery injury when the fixed nail of OLIF spreader is implanted [22]. In addition, neuroelectrophysiological monitoring should be performed in order to prevent severe nerve injury during OLIF surgery [6, 23].

Our study indicated that blood loss was less in the patients who undergone OLIF approach compared with that in the patients who undergone TLIF. The main reason is that OLIF approach avoids osteotomies. Thus, patients who undergone OLIF could recover faster than those undergone TLIF and would be discharged earlier. Furthermore, the taller cage of OLIF procedure increases the intervertebral distance and provides foraminal decompression without foraminotomy and can relieve lumbago, leg pain, or lower limb numbness better after surgery [24]. Our follow-up of 6 months after surgery showed that pain and functional scores, such as ODI and JOA, were improved more in the OLIF group than that in the TLIF group, which confirmed that the advantages of OLIF procedure were more in contrast to TLIF in the early stage of postoperation.

Our research has several advantages as follows. Specifically, we firstly reported the application of the concept of ERAS to OLIF and TLIF surgery by evaluating the change of blood indicators and postoperative symptom of patients. With this method, it will provide a better selective solution for clinical treatment. Moreover, objective scales to assess patient outcomes were generally used in previous studies with regard to the spine surgeries. However, it is limited to assess the perception of patients for their operation. Our study used patient postoperative satisfaction surveys to place more emphasis on patient perception, which also reflected the patient-centered treatment principles. The results of patient postoperative satisfaction survey displayed the treatment effect from a different perspective, which would present indirect guidance suggestions to the doctors that perioperative management included many aspects rather than surgery alone.

However, there are still some limitations in our study. As OLIF approach is a novel surgical approach of spine operation, the number of cases included is not sufficient. In the future, we will collect more cases and make subgroup analysis on the basis of specific diseases which are belonged to lumbar degenerative diseases. In addition, our research is a single-center study, and patients are mainly from eastern China. We can perform a multicenter study including the patients from different races and different districts.

## 5. Conclusions

This study indicated that OLIF and TLIF approaches were both rather effective therapies for patients with lumbar degenerative diseases. OLIF and TLIF approaches combined with ERAS program would not only provide the patients with minimally invasive spine surgery but also help them achieve satisfying and rapid recovery. Additionally, the effect of OLIF procedure could be better than TLIF procedure in the early stage after surgery. Therefore, the new idea of OLIF or TLIF approach combined with ERAS program may contribute to clinical practice.

## Figures and Tables

**Table 1:** Characteristics of the participants.

	TLIF	OLIF	*p* value
Age (years)	61.15 ± 5.52	61.84 ± 6.20	0.604
Gender			
Men (%)	57.5	55.3	0.842
BMI	22.20 ± 1.45	22.62 ± 1.60	0.233
Smokers (%)	27.5	21.1	0.507
Duration of operation (min)	102.45 ± 9.83	105.1 ± 8.93	0.217
Blood loss of operation (ml)	102.48 ± 14.22	59.53 ± 11.80	<0.001
Duration of hospital stay (days)	9.23 ± 0.95	7.87 ± 1.04	<0.001

OLIF: oblique lumbar interbody fusion; TLIF: transforaminal lumbar interbody fusion.

**Table 2:** Clinical outcome of the patients.

	Clinical outcome	Preoperation	Six months after surgery	Twelve months after surgery	*p* value
TLIF	VAS	7.35 ± 1.21	4.68 ± 1.21	2.38 ± 1.91	<0.001
	ODI (%)	44.38 ± 6.94	24.85 ± 3.86	14.82 ± 2.61	<0.001
	JOA	9.40 ± 3.18	19.40 ± 3.21	24.45 ± 1.87	<0.001
OLIF	VAS	7.55 ± 1.31	3.45 ± 1.54	1.76 ± 1.05	<0.001
	ODI (%)	46.95 ± 7.85	19.21 ± 4.20	13.55 ± 2.58	<0.001
	JOA	8.32 ± 2.42	22.21 ± 2.96	25.61 ± 2.02	<0.001

**Table 3:** Satisfaction of 12 months after surgery.

	TLIF	OLIF	*p* value
Very satisfied (%)	72.5	84.21	0.455
Satisfied (%)	22.5	13.16	
Dissatisfied (%)	5	2.63	

## Data Availability

The data used to support the findings of this study are included within the article.
